# Using Latent Representations to Link Disjoint Longitudinal Data for Mixed‐Effects Regression

**DOI:** 10.1002/sim.70701

**Published:** 2026-08-20

**Authors:** Clemens Schächter, Maren Hackenberg, Michelle Pfaffenlehner, Félix B. Tambe‐Ndonfack, Thorsten Schmidt, Astrid Pechmann, Janbernd Kirschner, Jan Hasenauer, Harald Binder

**Affiliations:** ^1^ Institute of Medical Biometry and Statistics (IMBI), Medical Faculty and Medical Center University of Freiburg Freiburg Germany; ^2^ Freiburg Center for Data Analysis, Modeling and AI University of Freiburg Freiburg Germany; ^3^ Department of Mathematical Stochastics, Mathematical Institute University of Freiburg Freiburg Germany; ^4^ Department of Neuropediatrics and Muscle Disorders, Medical Faculty and Medical Center University of Freiburg Freiburg Germany; ^5^ Bonn Center for Mathematical Life Sciences University of Bonn Bonn Germany; ^6^ Life and Medical Sciences (LIMES) Institute University of Bonn Bonn Germany; ^7^ CIBSS, Centre for Integrative Biological Signalling Studies University of Freiburg Freiburg Germany

**Keywords:** measurement instrument alignment, mixed‐effects regression, small data, spinal muscular atrophy, statistical inference, treatment switches, variational autoencoder

## Abstract

Many rare diseases offer limited established treatment options, leading patients to switch therapies when new medications emerge. To analyze the impact of such treatment switches within the low sample size limitations of rare disease trials, it is important to use all available data sources. This, however, is complicated when the use of measurement instruments changes during the observation period, for example when instruments are adapted to specific age ranges. The resulting disjoint longitudinal data trajectories complicate the application of traditional modeling approaches like mixed‐effects regression. We tackle this by mapping observations of each instrument to an aligned low‐dimensional temporal trajectory, enabling longitudinal modeling across instruments. Specifically, we employ a set of variational autoencoder architectures to embed item values into a shared latent space for each time point. Temporal disease dynamics and treatment switch effects are then captured through a mixed‐effects regression model applied to latent representations. To enable statistical inference, we present a novel statistical testing approach that accounts for the joint parameter estimation of mixed‐effects regression and variational autoencoders. The methodology is applied to quantify the impact of treatment switches for patients with spinal muscular atrophy. Here, our approach aligns motor performance items from different measurement instruments for mixed‐effects regression and maps estimated effects back to the observed item level to quantify the treatment switch effect. Our approach allows for model selection as well as for assessing effects of treatment switching. The results highlight the potential of modeling in joint latent representations for addressing small data challenges.

## 1 Introduction

Rare diseases often have a limited number of established treatments, leaving patients with few therapeutic options. Thus, when new medications emerge, patients often switch treatments. However, analyzing the impact of such treatment switches longitudinally is difficult since rare disease trials are often characterized by small data challenges [1, 2, 3]. These include small sample sizes, heterogeneous data collected across different sites and varying observation frequencies. Additional difficulties arise when the preferred instruments to measure clinical outcomes change during the observation window. Such changes can occur from an evolving gold standard or measurement instruments that are tailored to patient characteristics, like age or disease severity, that may change over time. However, using data from all available sources is important to make the most of the limited study population. For example, in children affected by spinal muscular atrophy (SMA), symptom progression is monitored longitudinally using several specialized, age‐ and symptom‐specific measurement instruments that evaluate different motor function skills [4, 5, 6, 7, 8, 9]. As patients age and their disease progresses, the preferred assessment instruments are adapted accordingly. Therefore, the longitudinal record often comprises only a subset of these instruments at a given time point.

Statistical approaches, such as mixed‐effects regression, are widely used for modeling disease progression in longitudinal data [10]. They estimate both population‐level effects (fixed effects) and subject‐specific deviations (random effects) simultaneously. Applications span multiple diseases where disease progression is measured via motor function or cognitive assessments. They include neuromuscular disorders such as SMA [11, 12, 13, 14], as well as central nervous system disorders such as Parkinson's disease [15, 16], and multiple sclerosis [17, 18]. Although use cases vary in these applications, we aim to model the impact of switching between medications using a mixed‐effects regression.

However, there are two core issues with the application to disjoint data sources as encountered in SMA data. First, multidimensional measurements are usually aggregated into a single score to reduce the model complexity, which discards the information contained in individual test items [19]. Second, handling switches in measurement instruments is often circumvented by limiting analysis to time frames where a specific measurement instrument was applied. However, this does not provide generality for the whole observation window, as patients with an improving or worsening disease condition can transition to a different measurement instrument to circumvent ceiling or floor effects at the top or bottom of the test scale [4, 9]. This introduces a bias, while a reduction in sample size reduces statistical power, which already is an issue in rare disease settings to begin with. While results of different studies reporting treatment effects are usually aggregated using a meta‐analysis [20, 21, 22], aligning results from different instruments is challenging due to within‐patient correlation across instruments and different measurement scales.

Motivated by these challenges in longitudinal data, we propose an approach that combines mixed‐effects regression with an artificial neural network approach, namely variational autoencoders (VAEs) [23]. We leverage VAEs to incorporate multiple measurement instruments at the item level, to obtain a latent representation at each time point of longitudinally observed data, while linking the time points by a mixed‐effects regression model. The aim is to facilitate modeling of treatment switch effects while providing an approach that allows for statistical inference on this joint low‐dimensional latent representation. To enable statistical testing, we introduce a bootstrap knockoff variable approach which corrects for potential biases caused by fitting the mixed‐effects model within a VAE architecture. As the mappings to and from the latent space are learnable and non‐linear, the approach can also compensate for ceiling effects present in the datasets. Therefore, a continuous outcome linear regression in the latent space is sufficient. To reflect different aspects of the patient characteristics measured by different instruments and allow for a flexible embedding, we employ a latent space with several dimensions, and correspondingly consider mixed models with a multivariate outcome.

The problem of extracting similar information from different measurement instruments has been considered to some extent in the field of item response theory [24], which is typically based on factor analysis [25, 26]. This includes linear and non‐linear factor models [27], and more general latent variable models [28, 29, 30]. Yet, these often rely on strong distributional assumptions and require anchor items present across different instruments [30], which limits applicability in settings with several clinical measurement instruments. The concept of domain adaptation, developed in the machine learning community, formalizes the challenge of linking data from a source and target domain under the assumption of a shared latent space. Applications have been particularly successful for image data [31, 32, 33], such as in MR imaging [34, 35] or microscopy [36]. There, deep learning approaches are employed to learn domain‐invariant representations [37, 38, 39]. These approaches generally assume a rather large number of observations. Also, they typically do not consider time structure, and when they do, focus on time series with a large number of time points. Therefore, while the general concept of domain adaptation might be useful for longitudinal rare disease registries, there is no readily applicable approach for small numbers of patients with limited observations. In particular, a combination of domain adaptation with statistical models for longitudinal models, such as mixed‐effects regression is missing so far. While VAEs have been combined with mixed‐effects models in the context of imaging data [40, 41] and intensive care unit data [42], this does not cover changes between measurement instruments. In our own work, we have previously combined VAEs with ordinary differential equations (ODEs) for modeling disease trajectories in a lower‐dimensional representation [43, 44, 45], based on a neural differential equation framework that allows for simultaneously fitting neural networks and dynamic models [46, 47, 48]. While we have extended this to also allow for two different measurement instruments [49], the focus on ODEs as deterministic models did not allow for statistical inference so far, as needed for statistically testing the effect of treatment switches. This could be provided by combining mixed‐effects regression within VAEs that align different measurement instruments.

The manuscript is structured as follows: First, we introduce the approach in Section 2, after a brief overview of VAEs and multivariate linear mixed‐effects models. To enable statistical testing, we introduce a bootstrap knockoff variable approach which characterizes and corrects for potential biases caused by fitting the mixed‐effects model on VAE obtained latent variables. This is accompanied by an approach for quantifying effects in the latent space by mapping them back to the item representation. In Section 3, we assess the approach using data for SMA patients from the SMArtCARE registry [50]. This registry reports observations collected using five different motor performance measurement instruments, which need to be integrated to analyze the impact of treatment switches. We compare the performance of the proposed approach to a method based on a meta‐analysis over per‐measurement instrument models. Additionally, we validate the approach by evaluating results when recovering artificially added treatment switches and illustrate how to perform model selection for the latent mixed‐effects model. A discussion is provided in Section 4, including a perspective on the potential of combining latent representations with statistical modeling for jointly analyzing data in small data settings. Finally, details on the hyperparameters, dataset, and model choice are given in the Supporting Information Section 1 and Tables are provided in Section Tables 1, 2, 3.

## 2 Methods

In the following, we denote random variables by italic uppercase letters (e.g., Y,Z), their realizations by bold lowercase letters (e.g., y,z), their corresponding (conditional) probability distributions by uppercase P with appropriate subscripts (e.g., $P_{Y},P_{Z|y}$), and their associated probability density functions by lowercase p (e.g., $p_{Y},p_{Z|y}$). Matrices are denoted by bold uppercase letters (e.g., Y,Z). Table 1 lists the notation used throughout the manuscript.

**TABLE 1:** Notation used throughout the manuscript.

Symbol	Meaning
i∈I	Patient index
$t\in T_{i},t\in T_{i,l}$	Observation times
$l\in L,l\in L_{i,t}$	Measurement instrument index
b∈B	Bootstrap samples
$n,n_{l}$	Data dimension
$m_{i},m_{i,l}$	Number of observations
d	Latent dimension
p,q	Number of fixed and random effect coefficients
rd	Difference in parameters between nested models
$Y,y_{i}/Z,z_{i}$	Single data/latent observation
$Y_{i},Y_{i}={\left(y_{i,t}\right)}_{t\in T_{i}}^{⊤}$	Data trajectory
$Z_{i},Z_{i}={\left(z_{i,t}\right)}_{t\in T_{i}}^{⊤}$	(Latent) response
$Y_{i,l},Y_{i,l}={\left(y_{i,t,l}\right)}_{t\in T_{i,l}}^{⊤}$	Instrument specific data trajectory
$Z_{i,l},Z_{i,l}={\left(z_{i,t,l}\right)}_{t\in T_{i,l}}^{⊤}$	Instrument specific latent trajectory
${\hat{Z}}_{i}={\left({\hat{z}}_{i,t}\right)}_{t\in T_{i}}^{⊤}$	Latent mixed model estimation
$X_{i},T_{i}$	Design matrices for fixed and random effects
$B,\hat{B}$	Fixed effect coefficient matrix
$U_{i},U_{i},{\hat{U}}_{i}$	Random effects
$E_{i},E_{i}$	Residual error
$\Phi ,\Sigma ,V_{i}$	Random effects covariance, residual covariance, precision
$I_{d}$	Identity matrix of dimension d
$P_{Y,Z},p_{Y,Z}(y,z)$	Joint generative distribution of (Y,Z)
$P_{Z},p_{Z}(z)$	Prior on latent variable
$P_{Y|z},p_{Y|z}(y)$	Conditional sampling distribution of Y given latent z
$P_{Y},p_{Y}(y)$	Marginal distribution of Y
$P_{Z|y},p_{Z|y}(z)$	True posterior of Z given y
$Q_{Z|y},q_{Z|y}(z)$	Variational posterior of Z given y
${\text{enc}}_{\phi },{\text{enc}}_{\phi_{l}},{\text{dec}}_{\theta },{\text{dec}}_{\theta_{l}}$	Encoder/decoder neural network
${ℒ}_{\text{VAE}}$	negative ELBO loss for VAE
${ℒ}_{\text{ML}},{ℒ}_{\text{REML}}$	(Restricted) ML log‐likelihood
${ℒ}_{\text{MMVAE}}$	Joint loss for multi‐measurement VAE + mixed model
β,η,γ	Weighting of KL and alignment terms in ${ℒ}_{\text{MMVAE}}$

### 2.1 VAEs

In this study, we use a set of variational autoencoder (VAE) [23] architectures to map data from the observation spaces of multiple measurement instruments into a shared low‐dimensional representation of patient characteristics and reconstruct them back to the original space. A variational autoencoder is a generative deep learning approach, that is, a model that allows for sampling, that learns latent representations of high‐dimensional data based on the principle of variational inference [51]. Therefore, a VAE provides tractable approximations to probability distributions without requiring restrictive distributional assumptions. In a first step, we introduce the classical VAE framework on a dataset containing i.i.d. observations from a single measurement instrument and time point, while we outline our approach to align multiple instruments and link them longitudinally via a mixed model in Section 2.3.

Consider i.i.d. observations ${\left\{y_{i}\right\}}_{i\in I},y_{i}\in {\mathbb{R}}^{n}$. A VAE introduces a latent variable $Z\in {\mathbb{R}}^{d}$ with d<n in a generative model $P_{Y,Z}$ to capture the underlying characteristics of the data. Specifically, the generative process draws a sample $z_{i}$ from a prior distribution $P_{Z}$, typically a multivariate standard normal $P_{Z}={𝒩}_{d}(0,I_{d})$, and then generates an observation $y_{i}$ from the conditional sampling distribution $P_{Y|z_{i}}$. Accordingly, the density of the joint distribution factorizes as $p_{Y,Z}(y_{i},z_{i})=p_{Y|z_{i}}(y_{i})p_{Z}(z_{i})$.

Within the VAE framework, the marginal likelihood of an observation, $p_{Y}(y)$, is obtained by

(1) $$\begin{array}{l} p_{Y}(y_{i})=\int_{{\mathbb{R}}^{d}}p_{Y|z}(y_{i})p_{Z}(z)dz. \end{array}$$

Therefore, evaluation of the marginal likelihood $p_{Y}(y_{i})$ involves integrating over the latent space, making the required computations analytically intractable. This intractability extends to the posterior $P_{Z|y_{i}}$, because Bayes' rule demands dividing by $p_{Y}(y_{i})$. As a result, any inference or model evaluation that relies on the exact posterior or marginal likelihood becomes unfeasible.

To circumvent this problem, VAEs employ a variational distribution $Q_{Z|y_{i}}\approx P_{Z|y_{i}}$, providing a tractable approximation to the true posterior. This variational distribution is chosen as a multivariate normal distribution with independent components, whose parameters are the function output of an encoder neural network ${\text{enc}}_{\phi }:{\mathbb{R}}^{n}\to {\mathbb{R}}^{d}\times {\mathbb{R}}_{>0}^{d}$ parameterized by learnable neural network parameters ϕ:

(2) $$\begin{array}{l} Q_{Z|y_{i}}={𝒩}_{d}(\mu_{i},\text{diag}(\sigma_{i}^{2}));\;(\mu_{i},\sigma_{i})={\text{enc}}_{\phi }(y_{i}). \end{array}$$

To enable gradient‐based optimization, latent values are obtained by sampling through the reparametrization trick, with decomposition

(3) $$\begin{array}{l} z_{i}=\mu_{i}+\sigma_{i}⊙\varepsilon_{i}, \end{array}$$

such that the gradient can be obtained from the non‐random part, while $\varepsilon_{i}$ is auxiliary noise drawn from a multivariate standard normal distribution ${𝒩}_{d}(0,I_{d})$, and ⊙ denotes the Hadamard product between two vectors. A decoder neural network ${\text{dec}}_{\theta }:{\mathbb{R}}^{d}\to \Psi$, parameterized by θ, maps the latent values back to the data space by modeling the parameters ψ∈Ψ of the conditional sampling distribution $P_{Y|z_{i}}$. The parameters of the encoder and decoder neural networks are simultaneously optimized by estimating and minimizing the evidence lower bound (ELBO) function

(4) $$\begin{array}{l} {ℒ}_{\text{VAE}}(\phi ,\theta )=-{𝔼}_{Z∼Q_{Z|y_{i}}}\left[\log p_{Y|z}(y_{i})\right]+\beta \text{KL}\left[Q_{Z|y_{i}}||P_{Z}\right], \end{array}$$

where KL[·||·] denotes the Kullback–Leibler (KL) divergence between the variational distribution $Q_{Z|y_{i}}$ and the prior $P_{Z}$ and the parameter β>0 balances the KL‐divergence with the reconstruction error term [52]. Optimization of the ELBO function is performed over mini‐batches, that is, randomly selected groups of observations, using stochastic gradient descent methods.

After estimating the encoder and decoder parameters, the VAE can be used to obtain a latent representation of each observation and to reconstruct the corresponding data‐level quantity. Specifically, for a data vector $y_{i}$, the encoder network maps it to the parameters of the posterior distribution $Q_{Z|y_{i}}$, which in the common Gaussian case yields as output ${\text{enc}}_{\phi }(y_{i})=(\mu_{i},\sigma_{i})$. After training a latent variable is obtained using the reparametrization trick (3), or alternatively via the posterior mean $z_{i}=\mu_{i}$ as a deterministic embedding of $y_{i}$. The decoder network then maps a latent representation back to the data space by specifying the parameters of the conditional distribution $P_{Y|z_{i}}$. A reconstruction of the original observation is obtained from the decoder output, either by drawing from the defined distribution or via the conditional mean.

For patients i∈I that have longitudinal measurements, we denote their data as $Y_{i}={\left(y_{i,t}\right)}_{t\in T_{i}}^{⊤}\in {\mathbb{R}}^{m_{i}\times n}$. The individual observations are collected at $m_{i}$ visit times $T_{i}=\{t_{i,1},\ldots ,t_{i,m_{i}}:0=t_{i,1}<\cdots <t_{i,m_{i}}\}$.

We optimize the network weights simultaneously using all $m_{i}$ visit‐level observations for each patient i∈I. As the longitudinal dependence across time points is modeled through a latent mixed‐effects regression, as described in the following section, and the VAE encoder only needs to be used for dimension reduction, which is the same task for each time point, simultaneous optimization of the VAE for all time points does not further take into account temporal dependency structure. The VAE encoder then yields for each patient i∈I, an $m_{i}\times d$‐dimensional representation $Z_{i}={\left(z_{i,t}\right)}_{t\in T_{i}}^{⊤}\in {\mathbb{R}}^{m_{i}\times d}$, which serves as the multivariate outcome in the mixed‐effects model.

### 2.2 Multivariate Longitudinal Mixed‐Effects Regression

To incorporate time structure into the latent representation, we utilize multivariate mixed‐effects regression models [53, 54]. They represent a patient trajectory as a realization of a random variable $Z_{i}\in {\mathbb{R}}^{m_{i}\times d}$, which is a linear combination of fixed effects, random effects, and residual errors:

(5) $$\begin{array}{l} Z_{i}=X_{i}B+T_{i}U_{i}+E_{i}. \end{array}$$

Fixed effects $B\in {\mathbb{R}}^{p\times d}$ with design matrix $X_{i}\in {\mathbb{R}}^{m_{i}\times p}$ represent the influence of p patient covariates with consistent effects across all patients. Such covariates could include age, sex, or administered medications. Interaction terms with age can model a change in influence over time.

Random effects $U_{i}\in {\mathbb{R}}^{q\times d}$ with design matrix $T_{i}\in {\mathbb{R}}^{m_{i}\times q}$ allow for individual‐specific deviations from the fixed group effects. Common choices include a random intercept and slope to model patient‐specific trajectories. In this study, we assume that the random effects follow a normal distribution $\text{vec}(U_{i})∼{𝒩}_{qd}(0,\Phi )$ with shared covariance matrix Φ across patients, where $\text{vec}:{\mathbb{R}}^{n\times m}\to {\mathbb{R}}^{nm}$ denotes the vectorization function, which stacks the columns of a matrix into a vector. Residual errors $E_{i}\in {\mathbb{R}}^{m_{i}\times d}$ account for variability not explained by fixed and random effects. Residuals are assumed to be independent across time given the random effects and across patients, $\text{Cov}\left(\text{vec}(U_{i}),\text{vec}(E_{i})\right)=0$. Furthermore, we assume that they follow a normal distribution $\text{vec}(E_{i})∼{𝒩}_{m_{i}d}(0,\Sigma ⊗I_{m_{i}})$, where ⊗ denotes the Kronecker product between two matrices.

The marginal distribution of the response variable $Z_{i}$ can be described as

(6) $$\begin{array}{l} \text{vec}(Z_{i})∼{𝒩}_{m_{i}d}\left(\text{vec}(X_{i}B),\underset{=:V_{i}^{-1}}{\underbrace{(I_{d}⊗T_{i})\Phi {\left(I_{d}⊗T_{i}\right)}^{⊤}+\Sigma ⊗I_{m_{i}}}}\right). \end{array}$$

Given observed patient trajectories $Z_{i}$ of the random variable $Z_{i}$, the variance parameters Φ and Σ are estimated using maximum likelihood (ML) or restricted maximum likelihood (REML) estimation [55], where the log‐likelihoods can be calculated as

(7a) $$\begin{array}{cc} & {ℒ}_{\text{ML}}\left(B,\Phi ,\Sigma \right)=\frac{1}{2}\underset{i\in I}{\sum }\\ & \;\left(\log\det(V_{i})-\text{vec}{\left(Z_{i}-X_{i}B\right)}^{⊤}V_{i}\;\text{vec}(Z_{i}-X_{i}B)-m_{i}d\log(2\pi )\right). \end{array}$$

(7b) $$\begin{array}{cc} & {ℒ}_{\text{REML}}\left(\Phi ,\Sigma \right)=\frac{1}{2}\underset{i\in I}{\sum }\\ & \;\left(\log\det(V_{i})-\log\det\left({\left(I_{d}⊗X_{i}\right)}^{⊤}V_{i}(I_{d}⊗X_{i})\right)\right.\\ & \;\left.-\text{vec}{\left(Z_{i}-X_{i}\hat{B}\right)}^{⊤}V_{i}\;\text{vec}(Z_{i}-X_{i}\hat{B})-(m_{i}-p)d\log(2\pi )\right). \end{array}$$

Optimization algorithms like Newton–Raphson or quasi‐Newton methods can be used to maximize the likelihood functions [56]. With an estimate of the covariance components and observed response $Z_{i}$, the realized best linear unbiased estimator (BLUE) $\hat{B}$ of the fixed effects, is given by

(8) $$\begin{array}{ll} \text{vec}(\hat{B}) & ={\left(\underset{i\in I}{\sum }{\left(I_{d}⊗X_{i}\right)}^{⊤}{\hat{V}}_{i}(I_{d}⊗X_{i})\right)}^{-1}\\ & \;\left(\underset{i\in I}{\sum }{\left(I_{d}⊗X_{i}\right)}^{⊤}{\hat{V}}_{i}\text{vec}(Z_{i})\right). \end{array}$$

The best linear unbiased predictor (BLUP) of the random effects $U_{i}$ can be obtained through

(9) $$\begin{array}{l} \text{vec}({\hat{U}}_{i})=\hat{\Phi }{\left(I_{d}⊗T_{i}\right)}^{⊤}{\hat{V}}_{i}\;\text{vec}(Z_{i}-X_{i}\hat{B}). \end{array}$$

### 2.3 Joining Multiple Measurement Instruments in a Latent Representation

**FIGURE 1:**
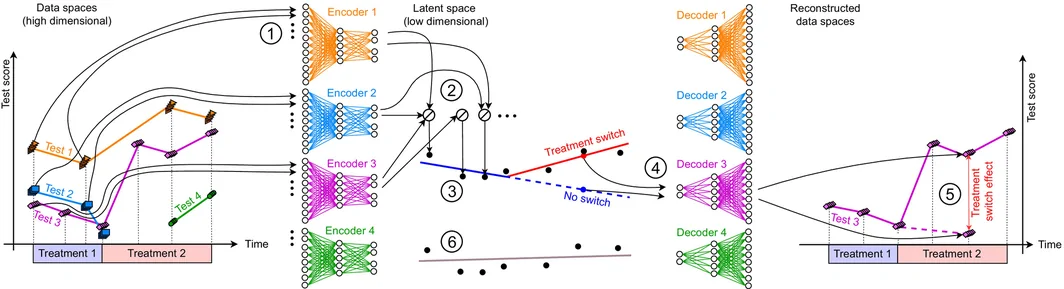
Schematic illustration of the proposed approach for multiple measurement instruments: (1) The items of each measurement instrument are encoded by a dedicated encoder network and corresponding latent values are drawn. (2) The latent values are averaged across instruments at each time step, to obtain a joint latent trajectory. (3) The averaged latent values serve as outcome variable for a multivariate mixed‐effects regression, which provides BLUE and BLUP estimators. (4) Predictions from the mixed‐effects model serve as input to a dedicated decoder network for each measurement instrument, for reconstruction at the original item level. (5) Treatment switch effects can be quantified per item. (6) A likelihood‐ratio test provides statistical inference.

For incorporating data from multiple measurement instruments l∈L⊂ℕ, where each instrument comprises $n_{l}$ test items, we use one encoder and decoder network per instrument, embedding data into a joint latent representation. Here observations are linked by multivariate mixed‐effects regression Equation (5), as schematically shown in Figure 1. We assume a patient i∈I is assessed using a subset of different measurement instruments at each time point. Denote by $T_{i,l}\subseteq T_{i}$ the observations in which measurement instrument l∈L was utilized. At the data level we obtain one trajectory per patient and observed instrument,

(10) $$\begin{array}{l} Y_{i,l}={\left(y_{i,t,l}\right)}_{t\in T_{i,l}}^{⊤}\in {\mathbb{R}}^{m_{i,l}\times n_{l}},\;l\in L, \end{array}$$

where $m_{i,l}$ denotes the overall number of observations for patient i with measurement instrument l. Measurement instrument specific encoder networks ${\text{enc}}_{\phi_{l}}$ are used to obtain the parameters of the variational distributions $Z_{l}∼Q_{Z_{l}|y_{i,t,l}}$ from which we draw d‐dimensional realizations

(11) $$\begin{array}{l} {\left(z_{i,t,l}\right)}_{t\in T_{i,l}}^{⊤}\in {\mathbb{R}}^{m_{i,l}\times d},\;l\in L. \end{array}$$

Denote with $L_{i,t}\subseteq L$ the index set of all measurement instruments used for patient i at observation time t. To obtain a unified latent trajectory for a patient, we average latent variables over all available values at the respective time points, yielding

(12) $$\begin{array}{l} Z_{i}={\left(z_{i,t}\right)}_{t\in T_{i}}^{⊤}={\left(\underset{l\in L_{i,t}}{\sum }\frac{z_{i,t,l}}{\left|L_{i,t}\right|}\right)}_{t\in T_{i}}^{⊤}\in {\mathbb{R}}^{m_{i}\times d}. \end{array}$$

We then model the longitudinal structure in the latent space using a multivariate mixed‐effects model of dimension d. Here, the mixed‐effects model provides the longitudinal link between visits while the VAEs provide the instrument‐specific measurement mappings to an aligned representation.

In particular, the latent mixed‐effects model can incorporate effects of treatment switches. Let $t_{{\text{switch}}_{i}}$ denote the time of a switch from one treatment to another. We collect the time differences relative to the switch

(13) $$\begin{array}{l} \Delta t_{i}={\left(t_{i,1}-t_{{\text{switch}}_{i}},t_{i,2}-t_{{\text{switch}}_{i}},\ldots ,t_{i,m_{i}}-t_{{\text{switch}}_{i}}\right)}^{⊤}. \end{array}$$

To capture group‐level trends following a treatment switch, the time since switch $\max(0,\Delta t_{i})$ is included as a fixed effect. To model individual deviations from group trends, there are random effects included for the time since $\max(0,\Delta t_{i})$ and the time up to a treatment switch $\min(0,\Delta t_{i})$. When multiple treatments are involved their influence can be separated into treatment specific covariates. Interaction terms with other covariates (e.g., age) can also be included in the mixed‐regression model. All decoder networks receive as input the prediction of the mixed model

(14) $$\begin{array}{l} {\hat{Z}}_{i}=X_{i}\hat{B}+T_{i}{\hat{U}}_{i} \end{array}$$

where $\hat{B}$ is the BLUE and ${\hat{U}}_{i}$ is the BLUP (see Equations (8) and (9)), given the latent outcome $Z_{i}$.

To estimate the parameters of the encoders and decoders as well as the latent mixed‐effects model, we use an iterative approach: To obtain the encoder and decoder parameters, $\left(\phi_{l},\theta_{l}\right)$ for l∈L, we freeze the mixed‐effect model parameters and minimize as a combination of per‐measurement instrument ELBO loss functions Equation (4) and two alignment terms over mini‐batches M⊂I:

(15) $$\begin{array}{cc} & {ℒ}_{\text{MMVAE}}\left({\left(\phi_{l},\theta_{l}\right)}_{l\in L}\right)\\ & \;=\frac{1}{\left|M|\sum_{i\in M}|T_{i}\right|}\left(\gamma \underset{i\in M}{\sum }\;\underset{t\in T_{i}}{\sum }\|{\hat{z}}_{i,t}-z_{i,t}{\|}_{2}^{2}-\eta {ℒ}_{\text{ML}}(B,\Phi ,\Sigma )\right)\\ & \;+\frac{1}{\left|M|\sum_{i\in M}\sum_{l\in L}|T_{i,l}\right|}\underset{i\in M}{\sum }\;\underset{l\in L}{\sum }\;\underset{t\in T_{i,l}}{\sum }\\ & \;\left(-{𝔼}_{Z_{l}∼Q_{Z_{l}|y_{l}}}\left[\log p_{Y_{l}|{\hat{z}}_{i,t}}\left(y_{i,t,l}\right)\right]+\beta \text{KL}\left[Q_{Z_{l}|y_{i,t,l}}||P_{Z}\right]\right). \end{array}$$

The first alignment term, weighted by η≥0 encourages agreement with the training criterion (ML/REML) of the frozen mixed model, while the second alignment term weighted by γ≥0, encourages agreement between the encoder output and the latent mixed model prediction.

Because the joint latent, serving as response to the mixed model, is the average of measurement specific latent variables $z_{i,t}=|L_{i,t}{|}^{-1}\sum_{l\in L_{i,t}}z_{i,t,l}$, the encoders are implicitly driven to place their outputs on a common scale. Instrument‐specific shifts or a rescaling inflates the disagreement penalty $\|{\hat{z}}_{i,t}-z_{i,t}{\|}_{2}^{2}$, as well as reduce the mixed‐effects likelihood, and as mixed model predictions are passed to the decoder ultimately the reconstruction loss and are therefore corrected during training.

After fitting the encoder and decoder parameters for given mixed‐effect model parameters for a given amount of iterations with a gradient descent method like Adam [57], a latent representation for each trajectory $Z_{i},i\in I$ is drawn. Afterwards, the mixed‐effects model is fitted using these latent representations as response, with ML or REML estimation (Equations (7a) and (7b)). Such alternating updates of the VAE and mixed‐effects model parameters are performed until the loss function ${ℒ}_{\text{MMVAE}}$ no longer steeply decreases and decreases saturate. We provide a pseudo‐code for this training procedure in Algorithm 1.

ALGORITHM 1 **Joint training procedure for the latent mixed model and VAEs.**

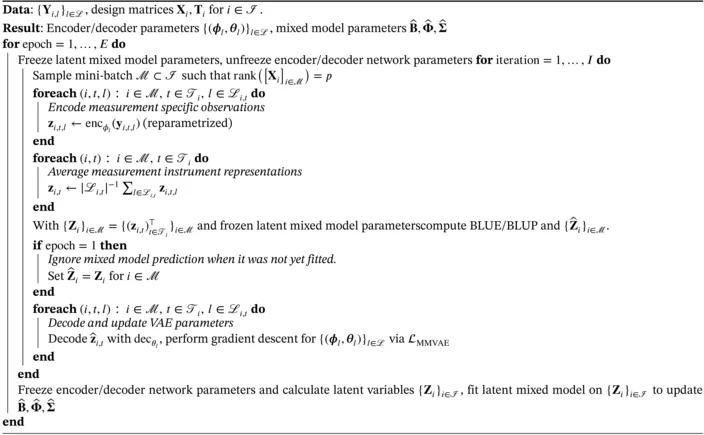

With the final parameter estimates, the impact of treatment switches can be quantified on the item level by decoding from the latent trajectory with and without treatment switch. These trajectories can be obtained by calculating the BLUE and BLUP, modifying the design matrices $X_{i},T_{i}$ by setting the covariates and interaction terms relating to time elapsed after treatment switches to zero and extending the covariates relating to time before treatment switches. The latent prediction of both models can be mapped back to the data level using the decoder neural networks and analyzed at the item level for differences.

### 2.4 Model Selection

Statistical testing based on the mixed‐effects model can be useful for further assessing the effect of treatment switches, as well as for potential model selection, for example, to decide which covariates to include for adjustment. One would normally assess the significance of a covariate by a likelihood‐ratio test [58] with test‐statistic $\Lambda \;=\;2({ℒ}_{\text{ML}\;\text{full}}-{ℒ}_{\text{ML}\;\text{red}})$. Here, ${ℒ}_{\text{ML}\;\text{full}}$ denotes the likelihood of a model containing the covariate of interest and ${ℒ}_{\text{ML}\;\text{red}}$ corresponds to the likelihood of a nested model without it. Under Wilks' theorem and standard regularity Λ is asymptotically $\chi_{rd}^{2}$ for a fixed effects block, with r being the number of tested covariates and d the dimension of the latent space.

The classical statistical model selection approach cannot be applied here as the final latent responses $Z_{i}$ depend on encoder parameters $\phi_{l},l\in L$ that were jointly optimized with the mixed‐effects coefficients. The same data is therefore used first to pick a representation and then to test a hypothesis, which can induce a post‐selection bias [59, 60]. In this setting the $\chi_{rd}^{2}$ reference distribution can be invalid. To correct for the intractable selection event (“the VAEs picked a particular latent representation”), we combine knockoff negative‐control covariates [61, 62] with bootstrap [59, 63]. The resulting procedure allows for inference for the joint VAE‐mixed‐effects estimator.

Let $w_{i}\in {\mathbb{R}}^{k}$ denote a k‐dimensional artificial knockoff covariate vector for individual i, where k is the number of generated negative‐control covariates. We assume the corresponding random variable $W_{i}$ is independent of ${\left\{(Z_{i},X_{i},T_{i})\right\}}_{i\in I}$ with $𝔼[W_{i}]=0,\;\text{Cov}(W_{i})=I_{k}$. For a subject with $m_{i}$ visits, these covariates are concatenated over visits as $W_{i}={\left(w_{i,1}\cdots w_{i,m_{i}}\right)}^{⊤}$, where $w_{i,j}$ is either identical across visits for patient‐level knockoffs or independently redrawn for visit‐level knockoffs.

We augment the fixed effect design matrix by ${\tilde{X}}_{i}=[X_{i}\;|W_{i}\;]$ and test

(16) $$\begin{array}{l} H_{0}:\;\beta_{W}=0\;\text{vs.}\;H_{1}:\;\beta_{W}\neq 0, \end{array}$$

where $\beta_{W}\in {\mathbb{R}}^{k\times d}$ denotes the coefficient block of $W_{i}$. Because $W_{i}⫫Z_{i}\;|\;X_{i},T_{i}$ by construction, $H_{0}$ is true with probability 1 and $W_{i}$ acts as a negative‐control predictor in the sense of the work by [61], yielding a reference statistic that captures what one should observe under the null hypothesis.

To empirically recreate the intractable selection event, we construct an empirical null distribution for the likelihood ratio (LR) test $F_{\Lambda }^{0}$ via bootstrap. For bootstrap samples b∈B, we randomly reinitialize the VAE parameters and regenerate knockoff variables $W_{i,b}$. Afterwards we re‐optimize for ${\left\{(\phi_{l,b},\theta_{l,b})\right\}}_{l\in L}$ to obtain latent responses $Z_{i,b},b\in B$, with which we calculate the LR statistic $\Lambda_{b}$ from the fitted full and reduced models. The empirical cumulative distribution function

(17) $$\begin{array}{l} {\hat{F}}_{\Lambda }^{0,B}(x)=\frac{1}{\left|B\right|}\underset{b\in B}{\sum }1\{\Lambda_{b}\leq x\} \end{array}$$

converges in probability to $F_{\Lambda }^{0}$ as |B|→∞, while resampling the knockoff together with the data (via different network weights) preserves the exchangeability property that underlies false discovery rate control in the original knockoff filter [62]. Consequently, the p value

(18) $$\begin{array}{l} \hat{p}=\frac{1+\sum_{b\in B}1\{\Lambda_{b}\geq \Lambda_{\text{obs}}\}}{|B|+1} \end{array}$$

is an unbiased estimate of ${\mathbb{P}}_{0}(\Lambda \geq \Lambda_{\text{obs}})$.

## 3 Results

We use the proposed approach on data from the SMArtCARE registry [50], which tracks the disease course of patients with SMA. SMA is a genetic disorder characterized by progressively declining motor function with symptom onset at birth or within early childhood. The severity of symptom progression is influenced by patient characteristics like genetic markers (e.g., count of the *SMN2* gene) or age at symptom onset and varies significantly across individuals [64]. Recently, there were advances in treating SMA [65], leading patients to switch to emerging treatments when they became available to them. Treatment assignment within the SMArtCARE registry is not randomized, yet mainly depends on the availability of a treatment in a medical center. To monitor the impact of treatment switches on disease development, longitudinal motor function assessments are conducted during semi‐regular visits. Here, movement ability is assessed using five specialized measurement instruments, the CHOP‐INTEND [7], HINE‐2 [8], HFMSE [6], RULM [5], and ALSFRS‐R [10] motor function tests. (See Table 2 for details.) In practice, infants and weak non‐sitters are evaluated with CHOP‐INTEND and motor milestone attainment in early childhood is captured with HINE‐2. When patients age, assessments transition to HFMSE to assess gross motor ability and RULM for upper‐limb performance, with ALSFRS‐R complementing motor scales in adult cohorts. Instrument changes often occur to use stage‐appropriate instruments and to avoid flooring and ceiling effects.

**TABLE 2:** Measurement instruments to assess a patient's motor function in SMArtCARE.

Full name	Abbr.	Test description	Details	
Revised upper limb module [5]	RULM	Measures upper limb function and strength across tasks like reaching, lifting, and hand movements.	**Target** **Median age** **Items** **Max score** **Inputs** **Obs.**	Children 11.1 20 + 1 37 + 6 63 3625
Hammersmith functional motor scale expanded [6]	HFMSE	Assesses gross motor skills, including activities like sitting, rolling, standing, and transitional movements.	**Target** **Median age** **Items** **Max score** **Inputs** **Obs.**	Children 9.6 33 + 1 66 + 6 104 3279
Children's Hospital of Philadelphia infant test of neuromuscular disorders [7]	CHOP‐INTEND	Assesses neuromuscular function, concentrating on spontaneous and prompted movements.	**Target** **Median age** **Items** **Max score** **Inputs** **Obs.**	Infants 3.0 16 64 68 1833
Hammersmith infant neurological examination, Section 2 [8]	HINE‐2	Monitors early development, especially the attainment of motor function milestones (sitting, standing, walking).	**Target** **Median age** **Items** **Max score** **Inputs** **Obs.**	Infants 4.4 8 + 3 26 +3 40 4071
ALS functional rating scale revised [10]	ALSFRS‐R	Covers speech, swallowing, and fine motor tasks; designed for ALS but also used for SMA.	**Target** **Median age** **Items** **Max score** **Inputs** **Obs.**	Adults 34.9 12 + 1 48 + 4 65 732

*Note:* Some tests contain additional items that are not used to determine the official test score. However, we still include these items to increase the amount of information.

Our approach integrates these five different motor function measurement instruments for a cohort of 522 patients, with a median of 17 individual observations per patient and mean of 2.1 out of 5 measurement instruments used per visit. The latent mixed‐effects regression model incorporates as covariates the time elapsed since treatment switch, age, a genetic marker, specifically the *SMN2* count, age at symptom onset, an indicator whether patients are currently asymptomatic for example because they were diagnosed via newborn screening, the current ventilation status, the current scoliosis status, sex of the patient and if a family member is affected by SMA. We also include interactions with age as fixed effects. Additionally, three random‐effect terms were used to allow for individual deviations from group‐level trends as random effects: a random intercept, and random slopes for the trajectory before and after the treatment switch.

After fitting our approach, we observed consistent alignment of similar motor function abilities across the five measurement instruments. We present these results in Supporting Information Figure S1.

### 3.1 Quantifying the Impact of Treatment Switches

A key question in the context of SMA treatment is the impact of treatment switches on the individual and the population level. To assess this effect in the light of different measurement approaches, we use the proposed approach to compare two sets of predicted disease trajectories (Equation (14)): one incorporating the treatment switch and a scenario in which the switch did not occur. We decoded the mixed‐effects model predictions from both scenarios for one year after the treatment change and compared their reconstructed motor function test item scores. The difference in total sum scores then served as our estimate of the treatment switch effect. This estimate should be interpreted as the net effect after treatment switch compared to the expected continuation of the previous treatment trajectory, meaning that the estimate does not necessarily separate the effect of the later treatment from potential carryover effects.

As seen in Table 3, there is a positive predicted improvement between 1.8% and 5.4% of the maximal score across the measurement instruments. Results are not deterministic as the encoder and decoder model distributions introduce additional stochasticity in the sampling and fitting process. However, assessed over ten random initializations there was no occurrence in which the aggregated treatment effect switched from positive to negative in one of the assessed instruments.

**TABLE 3:** Mean predicted item‐level differences for five motor function instruments (averaged over all patients and ten random seeds) between predictions with and without treatment switch one year after the switch took place.

Instrument	Unmodified data			Artificial +2		Artificial ×2	
	Mean	SD	Percentage	Mean	SD	Mean	SD
HINE‐2	0.87	0.20	3.00	2.84(+1.97)	0.57	1.35(×1.55)	0.18
RULM	1.47	0.12	3.41	2.62(+1.15)	0.41	1.58(×1.07)	0.32
CHOP	0.91	0.31	1.42	2.64(+1.73)	0.46	1.95(×2.14)	0.34
HFMSE	1.56	0.38	2.16	2.72(+1.16)	0.59	3.01(×1.92)	0.68
ALSFRS‐R	0.52	0.30	1.00	1.30(+0.78)	0.60	0.68(×1.31)	0.34

*Note:* Results are based on a three‐dimensional latent mixed effects trajectory. Positive values indicate improved motor function compared to no switch. The SD column lists the standard deviation of patient‐level mean values across the ten random seeds. The percentage column converts absolute differences to a percentage of the instrument's maximum sum‐score to enable cross‐instrument comparability. The left block refers to the switch detected in the unaltered data; the center to an artificial switch of +2 items per year; the right to an artificial doubling of the expected treatment switch.

To assess sensitivity to treatment‐related changes of known direction and approximate magnitude, we performed two semi‐synthetic perturbation analyses based on the observed SMA data. First, we simulated an additional artificial treatment effect by adding a sum‐score improvement of +2 points per year after treatment switch, distributed over randomly selected test items within each measurement instrument (see Appendix ? for details). This perturbation does not fully preserve ceiling effects, within‐observation item structure, or cross‐instrument alignment. We therefore expected the approach to detect a larger treatment‐switch effect compared with the original data, but to underestimate its magnitude. Consistent with this expectation, the estimated treatment‐switch effect increased by 0.78–1.97 items across measurement instruments, indicating correct directionality without overestimation of the imposed effect size. Second, we performed a complementary perturbation in which the covariates encoding time since treatment switch were multiplied by 0.5. Since, the same post‐switch trajectory is then represented on a time scale reduced by one half, the corresponding treatment‐switch coefficient is expected to increase approximately two‐fold. The model again recovered an increased treatment‐switch effect in the expected direction. Averaged across instruments, the estimated increase was approximately 1.6‐fold rather than the expected factor of two, indicating that the method recovered the imposed scaling but remained conservative in magnitude.

**FIGURE 2:**
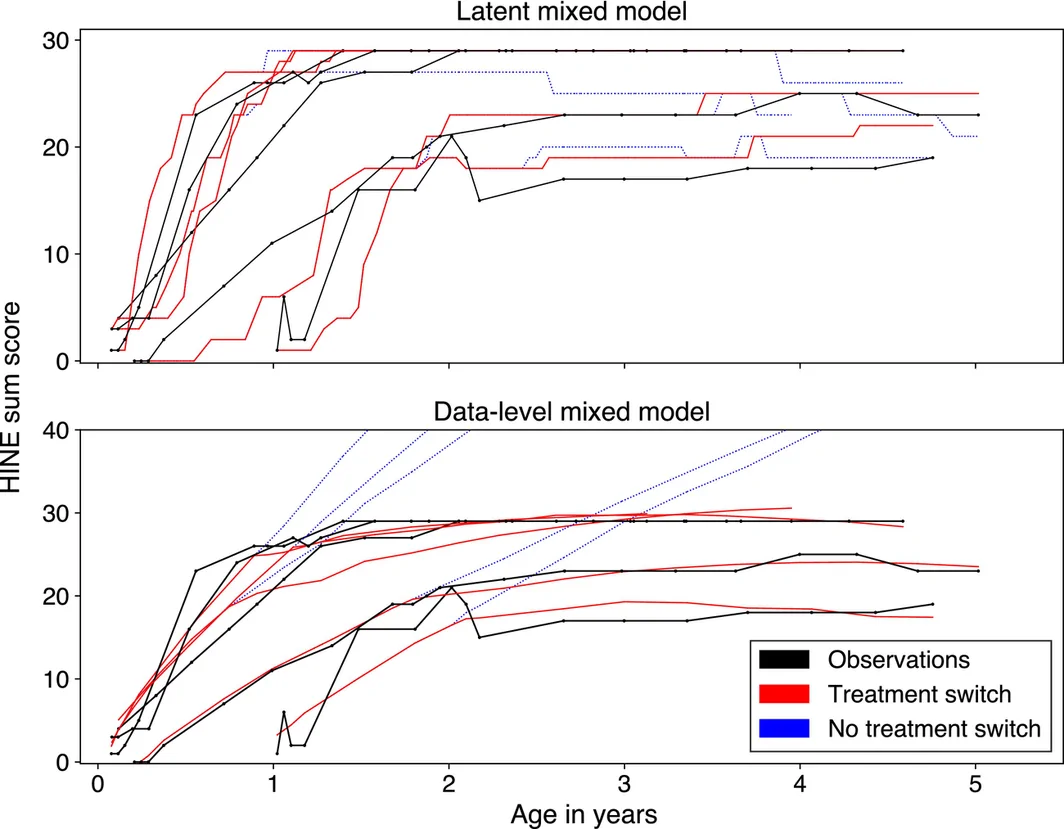
Comparison of total HINE‐2 sum scores for five patient trajectories. Observed data trajectories are displayed in black, the respective mixed model prediction with treatment switch in red, and mixed model predictions for a hypothetical trajectory without treatment switch in blue. The reconstructed trajectories of the latent mixed model are displayed in the upper subplot and for a standard data‐level mixed model in the lower subplot.

We also found that our approach reduces ceiling effects compared with a data‐level mixed model without additional parameters to account for ceiling or floor effects (see Figure 2). Because the linear latent mixed‐model predictions are transformed by decoder neural networks which models the data distribution with a ordinal distribution, samples fall naturally within a plausible score range. A similar effect within a data‐level approach would require additional parameters to model flooring and ceiling effects.

### 3.2 Latent Model Selection

We evaluated the statistical significance of effects in the mixed‐effects model, such as of treatment switches, with LR tests with critical values obtained from the proposed knockoff variable bootstrap procedure (Equation (17)). The empirical distribution of the test statistic was constructed with |B|=1000 bootstrap samples.

**FIGURE 3:**
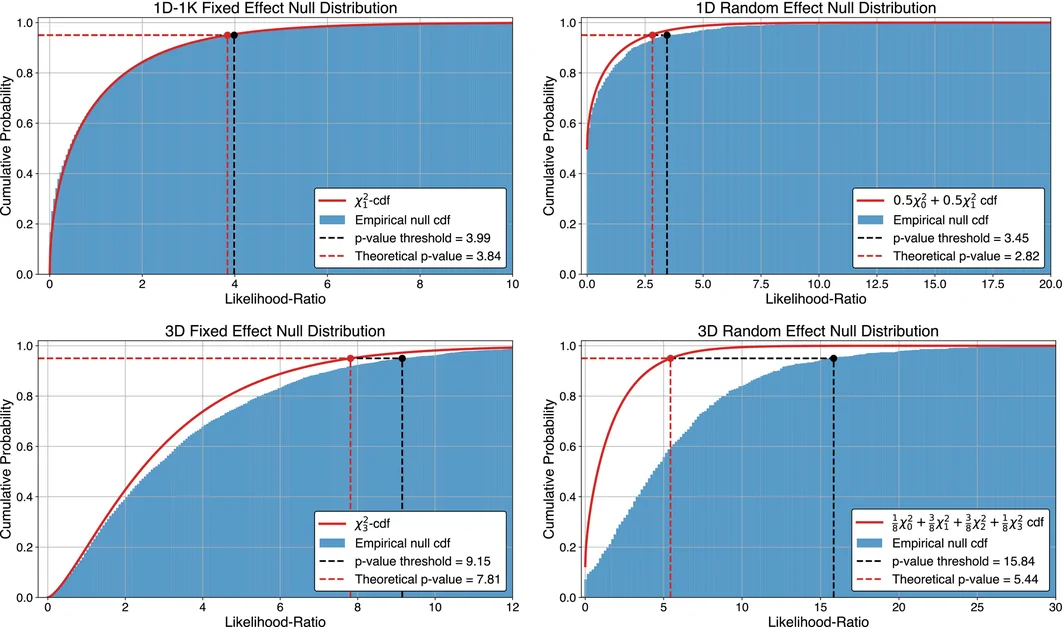
Empirical cumulative distribution functions of the null distributions of the likelihood ratio test statistic from artificially added knockoff variables for fixed (left column) and random effects (right column) in the mixed‐effects regression in latent representations of dimension d=1 (top row) and d=3 (bottom row). The red line represents the theoretical chi‐squared distribution that does not take into account the interdependent VAE and mixed model training procedure.

First, we compared the empirical null distributions with the theoretical $\chi^{2}$‐distributions that ignore the influence of the VAEs (see Figure 3). For a one‐dimensional latent representation, the empirical knockoff distribution follows the theoretical distribution closely. However, for the three‐dimensional representation that we want to use, there is a considerable difference, where the theoretical distribution would lead to an anti‐conservative test. This is seen for fixed effects as well as for random effects, where deviations are even larger.

Subsequently, we conducted LR tests for the 11 fixed effects covariates in the mixed‐effects model. We indicate with rd the degrees of freedom the theoretical $\chi^{2}$‐test statistic would follow, consisting of the amount of differences in included fixed effect (r) multiplied with the latent dimension (d). The effects for ventilation status ($\Lambda_{\text{obs}}=182.8,\text{SD}=28.0,rd=6$), and for treatment switches ($\Lambda_{\text{obs}}=335.2,\text{SD}=59.8,rd=12$), and disease onset ($\Lambda_{\text{obs}}=237.6,\text{SD}=29.7,rd=12$) and surgery for scoliosis ($\Lambda_{\text{obs}}=85.3,\text{SD}=14.36,rd=6$) showed high LR‐values, above critical values from the knockoff null distribution. Other covariates, such as sex ($\Lambda_{\text{obs}}=17.3,\text{SD}=5.4,rd=6$) and family history ($\Lambda_{\text{obs}}=11.2,\text{SD}=4.3,rd=6$), did not display significant effects.

As a further check, we generated data using the generative nature of the VAEs based on the fitted latent trajectories, but without the treatment switch effect, and refitted the mixed‐effects model to this newly generated dataset. The LR for including the treatment switch parameters dropped to 18.2, which no longer exceeded the empirical significance threshold (23.6 for rd=12, meaning d=3 latent dimensions with r=4 differences in covariates, consisting of switches to two SMA medications and their interactions with age).

### 3.3 Comparison With Naive Meta‐Analysis

As a comparison to the proposed approach, we fit separate linear mixed‐effects models for each motor instrument at the original data level, using the same fixed and random effects as in the latent model and taking instrument‐specific sum scores as outcomes. Additionally, we added an intercept to the fixed effects as test sum scores are not centered around zero compared to latent variables in our latent mixed model. Because many instruments are not observed with sufficient pre‐ and post‐switch visits for each patient, the effective sample is smaller than for the latent approach. Whereas the latent model could use a sample size of 522 patients, the data‐level models were restricted to 308 patients for the RULM measurement instrument, 318 for HINE‐2, 239 for HFMSE, and to 158 for CHOP‐INTEND. For the ALSFRS‐R test, too few patients (61) were available to fit a mixed model using the same set of covariates.

To pool treatment switch effects across instruments while accounting for overlapping patients, we performed a correlated‐effects fixed‐effect meta‐analysis via generalized least squares, using a patient‐level bootstrap to estimate the full cross‐instrument covariance and a bootstrap calibration for inference. For the combined treatment effects, the global likelihood‐ratio statistic was 214.06,rd=3 with a bootstrap‐calibrated *p* value of $p<10^{-3}$ (no exceedance out of 1000 null replicates). Therefore, the meta‐analysis provides strong evidence for a non‐zero treatment switch effect, consistent in magnitude with the latent mixed‐effects regression model.

When randomly sampled to a third of the original sample size, the latent approach could still reliably detect the treatment switch ($\Lambda_{\text{obs}}=102.5$), while the sample size was now often too small to get a stable fit for the data‐level approach, particularly for the CHOP‐INTEND measurement instrument.

## 4 Discussion

In clinical studies of rare diseases, it is essential to account for changing measurement strategies, for example, to assess treatment effects. As patient trajectories get longer and more observations become available, it becomes more likely that different measurement instruments are employed to monitor outcomes. This could be due to a changing state of the art or that measurement instruments are conditioned on patient characteristics. To exploit all historical data collected under different settings, novel methods are necessary. Here, we proposed an approach that combines VAEs with a latent longitudinal mixed‐effects regression model to capture disease progression and treatment switches in SMA data collected from multiple measurement instruments. By using encoder neural networks to map items from these instruments onto a unified latent representation, we handle systematic changes in measurement instruments during the observation period. To enable statistical inference, we introduced a knockoff variable bootstrap testing approach which allows us to correct for potential biases arising during model fitting.

This provides unique capabilities, which we demonstrated when assessing effects of treatment switches in patients with SMA. By mapping to a latent embedding via artificial neural networks, we utilize the item‐level details of observations. The learnable network mappings also handles ceiling effects and aligns measurement instruments of different scales. This allowed us to integrate observations of different measurement instruments, for example, tailored to different age ranges, to leverage a larger effective sample size compared to traditional methods that analyze each instrument separately. In other settings, power gains are particularly relevant for evaluating treatments in rare disease registries, where data is scarce. Potential bias in subsequent statistical inference, incurred by the flexibility of neural networks, could be mitigated by the proposed knockoff variable approach. At the same time, this approach allows for the detection of treatment switch effects in the SMA application, which might not have been identified otherwise.

Still there are several aspects that need to be carefully considered when utilizing the proposed approach. Although the proposed framework can reconstruct treatment‐related changes across different motor‐function instruments, the estimated treatment‐switch effects should be interpreted with caution. Unlike in classical parametric likelihood‐based models, the VAE component of our framework is primarily trained to learn a flexible latent representation with good reconstruction and generalization performance, rather than to recover uniquely identifiable data‐generating parameters. Consequently, the treatment‐switch coefficients should not be interpreted as exact quantitative parameter estimates in the classical sense, but as model‐based summaries of reconstructed treatment‐related patterns. The estimated treatment‐switch effects in our application should therefore be regarded as corresponding to approximate reconstructed patterns in the data, rather than direct estimates from a prespecified parametric model. The semi‐synthetic perturbation analyses suggest that externally imposed treatment‐related changes are recovered in the expected direction and with broadly consistent magnitude, although with some attenuation. Naturally, further validation is therefore needed before such estimates can be interpreted as quantitative treatment‐effect measures.

A related practical consideration is the choice of structural hyperparameters, in particular the latent dimension. While the projection to a low‐dimensional space reduces the number of required parameters, the latent dimensionality is still restricted to avoid over‐parametrization of the latent model, which can limit the modeled complexity of the underlying dynamics. On the other hand, choosing the latent dimension too large can increase the effects of overfitting, that is, a larger deviation of the skewed test statistic from the theoretical distribution. Thus, the number of latent dimensions needs to be carefully chosen. However, our knockoff‐bootstrap calibration accounts for the dependence between iteratively learning the latent representation and fitting the mixed‐effects regression, conditional on such structural hyperparameters being fixed a priori. Fixing such hyperparameters in the beginning, aligns with typical model selection strategies in biostatistics, for example, when fixing the number and position of knots in spline‐based regression before performing model selection with respect to non‐linear effects. Similar to such settings, model selection results have to be interpreted carefully as being conditional on initial choices. Generally, we recommend fixing structural hyperparameters based on clinical domain knowledge. When such knowledge is not available, there might be some data‐driven approaches that could still be useful, such as intrinsic‐dimension estimation for estimating the required latent dimension [66], or judging the reconstruction ability of a vanilla variational autoencoder fitted externally without the latent mixed‐effects model. In this case, one can first assess whether the chosen set of hyperparameters is able to capture the data patterns before linking observations by modeling disease progression with the proposed latent mixed‐effects model.

As datasets grow in complexity but not necessarily in size, which is typical for many rare diseases, there will be an increasing need for approaches that integrate deep learning methods and classical statistical theory. Such hybrid approaches can facilitate modeling on small but heterogeneous, high‐dimensional and multi‐modal datasets and provide answers for research questions that were previously challenging to address given the limited and complex data. As the explicit incorporation of statistical inference within deep learning frameworks is still uncommon, we therefore see this as a promising avenue of further research.

## 5 Software Implementation

We provide a software implementation of the proposed workflow in a GitHub repository. The repository contains a toy example illustrating the main components of the method, including training the mixed‐effects variational autoencoder, visualizing the learned latent representation, and testing fixed effects using an empirically calibrated null distribution.

## Funding

This work was supported by the Deutsche Forschungsgemeinschaft (DFG, German Research Foundation) Project‐ID 499552394 SFB 1597 Small Data.

## Conflicts of Interest

The authors declare no conflicts of interest.

## Supporting information




**Figure S1:** Latent alignment across five measurement instruments. In a one‐dimensional latent space, we decoded z‐values of a one‐dimensional model using each of the five instruments and plotted the expected sum scores of the resulting decoder distributions (see Figure 1). For each instrument, we marked attainment of motor‐function milestones when more than 50% of decoded samples achieved the respective milestone. Because items do not correspond one‐to‐one across instruments, we manually matched similar motor function items while disregarding slight deviations in their meaning. For instance, “rolling” in the ALSFRS‐R test reflects the ability to turn and roll in bed, whereas in HINE‐2 it encompasses rolling from prone to supine and vice versa over one side, and in the HFMSE test it encompasses being able to roll from supine to prone and vice versa over both sides. Overall, we observe a consistent alignment of motor‐function milestones across the different measurement scales.
**Table S1:** Reconstruction error statistics and estimated treatment switch effects for choices of the latent dimension d∈{1,2,3}. For smaller values of d, reconstruction quality, that is, the difference in test sum scores, decreases noticeably, while the estimated treatment effect remains unchanged in direction but becomes slightly smaller in magnitude.

## Data Availability

Real data cannot be shared due to legal and ethical restrictions.

## References

[1] A. A. Mitani and S. Haneuse , “Small Data Challenges of Studying Rare Diseases,” JAMA Network Open 3 (2020): e201965, 10.1001/jamanetworkopen.2020.1965. 32202640

[2] D. Whicher , S. Philbin , and N. Aronson , “An Overview of the Impact of Rare Disease Characteristics on Research Methodology,” Orphanet Journal of Rare Diseases 13 (2018): 14, 10.1186/s13023-017-0755-5. 29351763 PMC5775563

[3] C. M. W. Gaasterland , M. C. J. van der Weide , M. J. du Prie–Olthof , et al., “The Patient's View on Rare Disease Trial Design—a Qualitative Study,” Orphanet Journal of Rare Diseases 14 (2019): 31, 10.1186/s13023-019-1002-z. 30732630 PMC6367834

[4] E. S. Mazzone , A. Mayhew , J. Montes , et al., “Revised Upper Limb Module for Spinal Muscular Atrophy: Development of a New Module,” Muscle & Nerve 55 (2017): 869–874, 10.1002/mus.25430. 27701745

[5] J. M. O'Hagen , A. M. Glanzman , M. P. McDermott , et al., “An Expanded Version of the Hammersmith Functional Motor Scale for SMA II and III Patients,” Neuromuscular Disorders 17 (2007): 693–697, 10.1016/j.nmd.2007.05.009. 17658255

[6] A. M. Glanzman , E. Mazzone , M. Main , et al., “The Children's Hospital of Philadelphia Infant Test of Neuromuscular Disorders (CHOP INTEND): Test Development and Reliability,” Neuromuscular Disorders 20 (2010): 155–161, 10.1016/j.nmd.2009.11.014. 20074952 PMC3260046

[7] L. Haataja , E. Mercuri , R. Regev , et al., “Optimality Score for the Neurologic Examination of the Infant at 12 and 18 months of Age,” Journal of Pediatrics 135 (1999): 153–161, 10.1016/S0022-3476(99)70016-8. 10431108

[8] J. M. Cedarbaum , N. Stambler , E. Malta , et al., “The ALSFRS‐R: A Revised ALS Functional Rating Scale That Incorporates Assessments of Respiratory Function,” Journal of the Neurological Sciences 169 (1999): 13–21, 10.1016/S0022-510X(99)00210-5. 10540002

[9] R. Muni‐Lofra , G. Coratti , T. Duong , et al., “Assessing Disease Progression in Spinal Muscular Atrophy, Current Gaps, and Opportunities: A Narrative Review,” Neuromuscular Disorders 49 (2025): 105341, 10.1016/j.nmd.2025.105341. 40120531

[10] N. M. Laird and J. H. Ware , “Random‐Effects Models for Longitudinal Data,” Biometrics 38 (1982): 963–974, 10.2307/2529876. 7168798

[11] B. Cavaloiu , I. E. Simina , C. Vilciu , I. A. Trăilă , and M. Puiu , “Nusinersen Improves Motor Function in Type 2 and 3 Spinal Muscular Atrophy Patients Across Time,” Biomedicine 12 (2024): 1782, 10.3390/biomedicines12081782. PMC11351209 39200246

[12] P. Jacqmin , R. Gieschke , I. Delor , et al., “Mathematical Disease Progression Modeling in Type 2/3 Spinal Muscular Atrophy,” Muscle & Nerve 58 (2018): 528–535, 10.1002/mus.26178. 29938801

[13] T. Duong , C. Wolford , M. McDermott , et al., “Nusinersen Treatment in Adults With Spinal Muscular Atrophy,” Neurology: Clinical Practice 11 (2021): e317–e327, 10.1212/CPJ.0000000000001033. 34476123 PMC8382360

[14] M. Oskoui , J. W. Day , N. Deconinck , et al., “Two‐Year Efficacy and Safety of Risdiplam in Patients With Type 2 or Non‐Ambulant Type 3 Spinal Muscular Atrophy (SMA),” Journal of Neurology 270 (2023): 2531–2546, 10.1007/s00415-023-11560-1. 36735057 PMC9897618

[15] A. M. Hanff , C. McCrum , A. Rauschenberger , et al., “Sex‐Specific Progression of Parkinson's Disease: A Longitudinal Mixed‐Models Analysis,” Journal of Parkinson's Disease 15 (2025): 805–818, 10.1177/1877718X251339201. PMC13347478 40388933

[16] S. A. Miller , M. Mayol , E. S. Moore , A. Heron , V. Nicholos , and B. Ragano , “Rate of Progression in Activity and Participation Outcomes in Exercisers With Parkinson's Disease: A Five‐Year Prospective Longitudinal Study,” Parkinson's Disease 2019 (2019): 5679187, 10.1155/2019/5679187. PMC6778930 31662843

[17] A. Bralić , S. Pavelin , N. Pleić , et al., “Correspondence of MRI and nTMS With EDSS in Multiple Sclerosis: Longitudinal Follow‐Up Study,” Annals of Clinical Translational Neurology 12 (2025): 1240–1255, 10.1002/acn3.70041. 40244940 PMC12172135

[18] T. I. Lin and J. C. Lee , “A Robust Approach to t Linear Mixed Models Applied to Multiple Sclerosis Data,” Statistics in Medicine 25 (2006): 1397–1412, 10.1002/sim.2384. 16220509

[19] D. McNeish and M. G. Wolf , “Thinking Twice About Sum Scores,” Behavior Research Methods 52 (2020): 2287–2305, 10.3758/s13428-020-01398-0. 32323277

[20] C. Pascual‐Morena , V. Martínez‐Vizcaíno , I. Cavero‐Redondo , et al., “Efficacy of Risdiplam in Spinal Muscular Atrophy: A Systematic Review and Meta‐Analysis,” Pharmacotherapy 44 (2024): 97–105, 10.1002/phar.2866. 37574770

[21] T. Hagenacker , L. Maggi , G. Coratti , et al., “Effectiveness of Nusinersen in Adolescents and Adults With Spinal Muscular Atrophy: Systematic Review and Meta‐Analysis,” Neurology and Therapy 13 (2024): 1483–1504, 10.1007/s40120-024-00653-2. 39222296 PMC11393259

[22] D. P. Kingma and M. Welling , “Auto‐Encoding Variational Bayes,” 2013 arXiv Preprint, 10.48550/arXiv.1312.6114.

[23] M. J. Kolen and R. L. Brennan , Test Equating, Scaling, and Linking: Methods and Practices (Springer, 2014), 10.1007/978-1-4939-0317-7.

[24] R. J. Wirth and M. C. Edwards , “Item Factor Analysis: Current Approaches and Future Directions,” Psychological Methods 12 (2007): 58–79, 10.1037/1082-989X.12.1.58. 17402812 PMC3162326

[25] A. Skrondal and S. Rabe‐Hesketh , Generalized Latent Variable Modeling: Multilevel, Longitudinal, and Structural Equation Models (Chapman and Hall/CRC, 2004), 10.1201/9780203489437.

[26] D. J. Bauer and A. M. Hussong , “Psychometric Approaches for Developing Commensurate Measures Across Independent Studies: Traditional and New Models,” Psychological Methods 14 (2009): 101–125, 10.1037/a0015583. 19485624 PMC2780030

[27] M. J. Kolen , “Population Invariance in Equating and Linking: Concept and History,” Journal of Educational Measurement 41 (2004): 3–14, 10.1111/j.1745-3984.2004.tb01155.x.

[28] S. Van Buuren , S. Eyres , A. Tennant , and M. Hopman‐Rock , “Improving Comparability of Existing Data by Response Conversion,” Journal of Official Statistics 21 (2005): 53–72.

[29] E. R. van den Heuvel , L. E. Griffith , N. Sohel , I. Fortier , G. Muniz‐Terrera , and P. Raina , “Latent Variable Models for Harmonization of Test Scores: A Case Study on Memory,” Biometrical Journal 62 (2020): 34–52, 10.1002/bimj.201800146. 31583767

[30] G. Csurka , “A Comprehensive Survey on Domain Adaptation for Visual Applications,” in Domain Adaptation in Computer Vision Applications, ed. G. Csurka (Springer, 2017), 1–35, 10.1007/978-3-319-58347-1_1.

[31] Y. Ganin and V. Lempitsky , “Unsupervised Domain Adaptation by Backpropagation,” in Proceedings of the 32nd International Conference on Machine Learning, vol. 37 (PMLR, 2015), 1180–1189.

[32] H. Guan and M. Liu , “Domain Adaptation for Medical Image Analysis: A Survey,” IEEE Transactions on Biomedical Engineering 69 (2022): 1173–1185, 10.1109/TBME.2021.3117407. 34606445 PMC9011180

[33] M. Götz , C. Weber , F. Binczyk , et al., “DALSA: Domain Adaptation for Supervised Learning From Sparsely Annotated MR Images,” IEEE Transactions on Medical Imaging 35 (2016): 184–196, 10.1109/TMI.2015.2463078. 26259241

[34] M. Ghafoorian , A. Mehrtash , T. Kapur , et al., “Transfer Learning for Domain Adaptation in MRI: Application in Brain Lesion Segmentation,” in Medical Image Computing and Computer Assisted Intervention—MICCAI 2017, LNCS 10435 (Springer International Publishing, 2017), 516–524, 10.1007/978-3-319-66179-7_59.

[35] C. Becker , C. M. Christoudias , and P. Fua , “Domain Adaptation for Microscopy Imaging,” IEEE Transactions on Medical Imaging 34 (2015): 1125–1139, 10.1109/TMI.2014.2376872. 25474809

[36] M. Chen , Z. Xu , K. Q. Weinberger , and F. Sha , “Marginalized Denoising Autoencoders for Domain Adaptation,” in International Conference on Machine Learning 2012 (Omnipress, 2012), 767–774.

[37] M. Long , Y. Cao , J. Wang , and M. I. Jordan , “Learning Transferable Features With Deep Adaptation Networks,” in Proceedings of the 32nd International Conference on Machine Learning, vol. 37 (PMLR, 2015), 1180–1189, 10.1145/2783258.2788606.

[38] E. Tzeng , J. Hoffman , K. Saenko , and T. Darrell , “Adversarial Discriminative Domain Adaptation,” in 2017 IEEE Conference on Computer Vision and Pattern Recognition (CVPR) (IEEE, 2017), 2962–2971, 10.1109/CVPR.2017.316.

[39] R. Couronné , P. Vernhet , and S. Durrleman , “Longitudinal Self‐Supervision to Disentangle Inter‐Patient Variability From Disease Progression,” in Medical Image Computing and Computer Assisted Intervention—MICCAI 2021 Lecture Notes in Computer Science, vol. 12902 (Springer International Publishing, 2021), 231–241, 10.1007/978-3-030-87196-3_22.

[40] B. Sauty and S. Durrleman , “Progression Models for Imaging Data With Longitudinal Variational Auto Encoders,” in Medical Image Computing and Computer Assisted Intervention—MICCAI 2022 (Springer Nature Switzerland AG, 2022), 3–13, 10.1007/978-3-031-16431-6_1.

[41] P. Ong , M. Haußmann , O. Lönnroth , and H. Lähdesmäki , “Latent Mixed‐Effect Models for High‐Dimensional Longitudinal Data,” 2024 arXiv Preprint, 10.48550/arXiv.2409.11008.

[42] G. Köber , S. Pooseh , H. Engen , et al., “Individualizing Deep Dynamic Models for Psychological Resilience Data,” Scientific Reports 12 (2022): 8061, 10.1038/s41598-022-11650-6. 35577829 PMC9110739

[43] M. Hackenberg , A. Pechmann , C. Kreutz , J. Kirschner , and H. Binder , “A Statistical Approach to Latent Dynamic Modeling With Differential Equations,”. The American Statistician (2026), 10.1080/00031305.2025.2539999.

[44] M. Hackenberg , P. Harms , M. Pfaffenlehner , et al., “Deep Dynamic Modeling With Just Two Time Points: Can We Still Allow for Individual Trajectories?,” Biometrical Journal 64 (2022): 1426–1445, 10.1002/bimj.202000366. 35384018

[45] Y. Rubanova , R. T. Q. Chen , and D. K. Duvenaud , “Latent ODEs for Irregularly‐Sampled Time Series,” 2019 arXiv Preprint, 10.48550/arXiv.1907.03907.

[46] V. Fortuin , D. Baranchuk , G. Rätsch , and S. Mandt , “GP‐VAE: Deep Probabilistic Time Series Imputation,” in International Conference on Artificial Intelligence and Statistics, vol. 108 (PMLR, 2020), 1651–1661.

[47] A. Alaa , E. Mayer , and M. Barahona , “ICE‐NODE: Integration of Clinical Embeddings With Neural Ordinary Differential Equations,” in Machine Learning for Healthcare Conference 2022 (PMLR, 2022), 537–564.

[48] M. Hackenberg , M. Pfaffenlehner , M. Behrens , A. Pechmann , J. Kirschner , and H. Binder , “Investigating a Domain Adaptation Approach for Integrating Different Measurement Instruments in a Longitudinal Clinical Registry,” 2023 arXiv Preprint, 10.48550/arXiv.2312.00616. PMC11656297 39698740

[49] A. Pechmann , K. König , G. Bernert , et al., “SMArtCARE—a Platform to Collect Real‐Life Outcome Data of Patients With Spinal Muscular Atrophy,” Orphanet Journal of Rare Diseases 14 (2019): 18, 10.1186/s13023-019-0998-4. 30665421 PMC6341722

[50] D. M. Blei , A. Kucukelbir , and J. D. McAuliffe , “Variational Inference: A Review for Statisticians,” Journal of the American Statistical Association 112 (2017): 859–877, 10.1080/01621459.2017.1285773.

[51] I. Higgins , L. Matthey , A. Pal , et al., “beta‐VAE: Learning Basic Visual Concepts with a Constrained Variational Framework,” in International Conference on Learning Representations (OpenReview.net, 2017), https://openreview.net/forum?id=Sy2fzU9gl.

[52] J. O. Chipperfield and D. G. Steel , “Multivariate Random Effect Models With Complete and Incomplete Data,” Journal of Multivariate Analysis 109 (2012): 146–155, 10.1016/j.jmva.2012.02.014.

[53] Y. Amemiya , “On Multivariate Mixed Model Analysis,” IMS Lecture Notes‐Monograph Series, vol. 24., CA: Institute of Mathematical Statistics, 10.1214/lnms/1215463787.

[54] J. C. Pinheiro and D. M. Bates , Mixed‐Effects Models in S and S‐PLUS: Methods and Practices (Springer, 2000), 10.1007/b98882.

[55] I. N. Latra , S. Linuwih , Purhadi , and Suhartono , “Estimation for Multivariate Linear Mixed Models,” International Journal of Basic and Applied Sciences 10 (2010): 79–86.

[56] D. P. Kingma and J. Ba , “Adam: A Method for Stochastic Optimization,” 2014 arXiv Preprint, 10.48550/arXiv.1412.6980.

[57] S. S. Wilks , “The Large‐Sample Distribution of the Likelihood Ratio for Testing Composite Hypotheses,” Annals of Mathematical Statistics 9 (1938): 60–62, 10.1214/aoms/1177732360.

[58] J. Taylor and R. J. Tibshirani , “Statistical Learning and Selective Inference,” Proceedings of the National Academy of Sciences 112 (2015): 7629–7634, 10.1073/pnas.1507583112. PMC4485109 26100887

[59] J. D. Lee , D. L. Sun , Y. Sun , and J. E. Taylor , “Exact Post‐Selection Inference, With Application to the Lasso,” Annals of Statistics 44 (2016): 907–927, 10.1214/15AOS1371.

[60] R. F. Barber and E. J. Candès , “Controlling the False Discovery Rate via Knockoffs,” Annals of Statistics 43 (2015): 2055–2085, 10.1214/15-AOS1337.

[61] T. B. Nguyen , J. A. Chevalier , B. Thirion , and S. Arlot , “Aggregation of Multiple Knockoffs,” in International Conference on Machine Learning 2020, vol. 119 (PMLR, 2020), 7283–7293.

[62] B. Efron , “Bootstrap Methods: Another Look at the Jackknife,” Annals of Statistics 7 (1979): 1–26, 10.1214/aos/1176344552.

[63] S. J. Kolb and J. T. Kissel , “Spinal Muscular Atrophy: A Timely Review,” Archives of Neurology 68 (2011): 979–984, 10.1001/archneurol.2011.74. 21482919 PMC3860273

[64] C. J. J. Yeo , E. F. Tizzano , and B. T. Darras , “Challenges and Opportunities in Spinal Muscular Atrophy Therapeutics,” Lancet Neurology 23 (2024): 205–218, 10.1016/S1474-4422(23)00419-2. 38267192

[65] E. Levina and P. J. Bickel , “Maximum Likelihood Estimation of Intrinsic Dimension,” in Advances in Neural Information Processing Systems (MIT Press, 2005), 17.

[66] E. Facco , M. d'Errico , A. Rodriguez , and A. Laio , “Estimating the Intrinsic Dimension of Datasets by a Minimal Neighborhood Information,” Scientific Reports 7 (2017): 12140. 28939866 10.1038/s41598-017-11873-y PMC5610237

