# Supplementary material for: Using Latent Representations to Link Disjoint Longitudinal Data for Mixed‐Effects Regression
Source: Stat Med. 2026 Aug 20;45(18-19):e70701. doi: 10.1002/sim.70701 (PMC13491306; doi:10.1002/sim.70701)
Supplement: Supplementary file 1 [file SIM-45-0-s001.pdf]

Supporting information for "Using latent representations to link disjoint longitudinal data for mixed-effects regression"

Clemens Schächter[1,2,*], Maren Hackenberg[1,2], Michelle Pfaffenlehner[1,2], Félix B. Tambe-Ndonfack[3], Thorsten Schmidt[3], Astrid Pechmann[4], Janbernd Kirschner[4], Jan Hasenauer[5,6], and Harald Binder[1,2,7]

[1]Institute of Medical Biometry and Statistics (IMBI), Medical Faculty and Medical Center – University of Freiburg, Germany
[2]Freiburg Center for Data Analysis, Modeling and AI – University of Freiburg, Germany
[3]Department of Mathematical Stochastics, Mathematical Institute – University of Freiburg, Germany
[4]Department of Neuropediatrics and Muscle Disorders, Medical Faculty and Medical Center – University of Freiburg, Germany
[5]Bonn Center for Mathematical Life Sciences – University of Bonn, Germany
[6]Life and Medical Sciences (LIMES) Institute – University of Bonn, Germany
[7]CIBSS, Centre for Integrative Biological Signalling Studies – University of Freiburg, Germany
[*]Corresponding author, contact: clemens.schaechter@uniklinik-freiburg.de

May 21, 2026

1 Data preprocessing and Hyperparameters

In collaboration with our clinical partners from the SMArtCARE registry, we restricted our analysis to patients who underwent a treatment switch. To define this cohort, we filtered these patients based on the following criteria. First, patients who received less than six months of treatment with a medication before or after the switch were excluded. For patients who switched medication more than twice, we included their data only up to the second treatment switch. Further, we required that patients had motor-function evaluations at a minimum of four visits, including at least two visits before and after the switch. Lastly, we excluded

switches to medications with fewer than 10 patients, which left the dataset with switches to two different SMA medications.

This resulted in 522 patients with an average of 17.3 hospital visits and a mean of 2.1 out of 5 tests used to evaluate motor function at each visit. The median period under observation period was 5.7 years, while the median patient age was 6.7 years.

We binarized each test item using thermometer encoding, since physicians grade motor function on ordinal categorical scales comprising between two and six levels per test item. We also included an indicator variable for each motor function item that was set to one if a test item could not be performed by a patient. To capture the data distribution, we chose an ordinal logistic model as decoder distribution [1].

Missing values within an individual assessment were imputed by zero-filling, reflecting the judgment of our clinical partners that such items were typically skipped due to patient exhaustion. However, observations with more than a quarter of missing values were excluded from analysis, as some child patients occasionally refuse to perform a larger set of tasks due to bad mood despite being physically able to perform them.

Some covariates were split up into multiple fixed effects; these included the *SMN2* count, which was one-hot encoded into two categories (*SMN2* ≤ 2 and *SMN2* ≥ 3). Age at symptom onset was divided into a continuous variable representing the age at symptom onset in years for symptomatic patients and an indicator variable showing whether a patient was still presymptomatic. Years since treatment switch was divided into two separate variables each indicating the years since switching to medication A and B. We standardized the covariates except time since medication switch and included interaction terms with age for all fixed effects (except age itself). We included an intercept for data-level mixed models, but not in the latent model as latent variables are centered around zero to follow the standard normal prior distribution.

We chose dense neural networks consisting of two hidden layers with 250 and 100 units and $\tanh$ activation function within encoder and decoder network architectures. Architectural choices were obtained by pre-fitting a standard VAE without longitudinal component on the datasets and monitoring the reconstruction ability. We found the model to be relatively insensitive to the choice of network architecture and decided not to tune for this parameter. We trained neural networks with the Adam optimizer [2] with standard hyperparameters and mixed models using the L-BFGS algorithm with $\eta = 0.15$ [3]. In each epoch we updated the

VAE parameters 100 times on the whole dataset before refitting the mixed model parameters. We trained for 20 epochs, after which we observed no further improvement in total loss and reconstruction quality for more than two epochs.

In the small-data setting considered, a single training run of the proposed method was computationally inexpensive and could be completed within around two minutes on an Apple M2 Max processor (3.49 GHz, 16 cores). Consequently, obtaining the bootstrap null distribution with 1000 samples took around 1.5 days. In general, the runtime of a single training rum depends on the dataset size, the number of observations per subject, the number of measurement instruments, and the dimensionality of the data and latent space.

The latent space dimensionality was set to $d = 3$, chosen by domain knowledge to be expressive enough for three aspects of SMA. First, signal forwarding, meaning the short-term efficiency of neuromuscular transmission, second motor neuron integrity, reflecting the slowly varying capacity of the motor unit pool, and lastly the development of motor function. As d might be a critical parameter, we complemented our choice of this parameter based on domain analysis by investigating the impact of different candidate values of d close to our knowledge-based choice (in Supporting Information, Table S1). There, we found treatment-switch effect estimates to be rather robust. For settings where d cannot be chosen based on clinical knowledge, unfortunately there is no established purely data-driven approach in VAE community. Still, there are some criteria that might be useful. For example, one could estimate the intrinsic dimensionality of the dataset, which attempts to quantify the effective number of latent variables required to represent the dominant data patterns [4, 5]. Alternatively, an architecture choice could be obtained by fitting a standard VAE on a train/test split, while recognizing that the latent dimension is constrained by the available sample size and parameter count of the latent mixed model.

In the loss function (Eq. 15), we down-weighted the KL divergence loss term by 0.5 because of the modest data dimensionality which led to an impact of reconstruction quality for higher KL-weighting. The weighted KL term converged to contribute roughly 10% to the loss. The alignment terms were up-weighted by 5 and converged to contribute about 2.5% each to the final loss, placing primary emphasis on reconstruction quality. Model performance remained stable when these coefficients were halved or doubled. Increasing the alignment terms tended to increase skewness of the empirical test-statistic distribution, whereas decreasing them led to larger deviations between encoder outputs and latent mixed-model predictions. We chose a diagonal covariance structure for $\mathbf{\Phi}, \mathbf{\Sigma}$. We found that

alternative covariance structures did not contribute to a better reconstruction quality of test items, while conditional residuals were approximately uncorrelated.

To simulate an artificial treatment effect, we first computed the incremental score to be added for each observation; no extra score was added to measurements before the treatment switch, after the switch an artificial improvement of one item every six months per test was applied. This increment was implemented by randomly selecting test items for each measurement instrument that had not yet reached their maximum value and increasing their score by one for each subsequent observation. If an item was already at its full score in one of these subsequent observations, the additional points were allocated to another randomly chosen item if possible.

2 Additional results

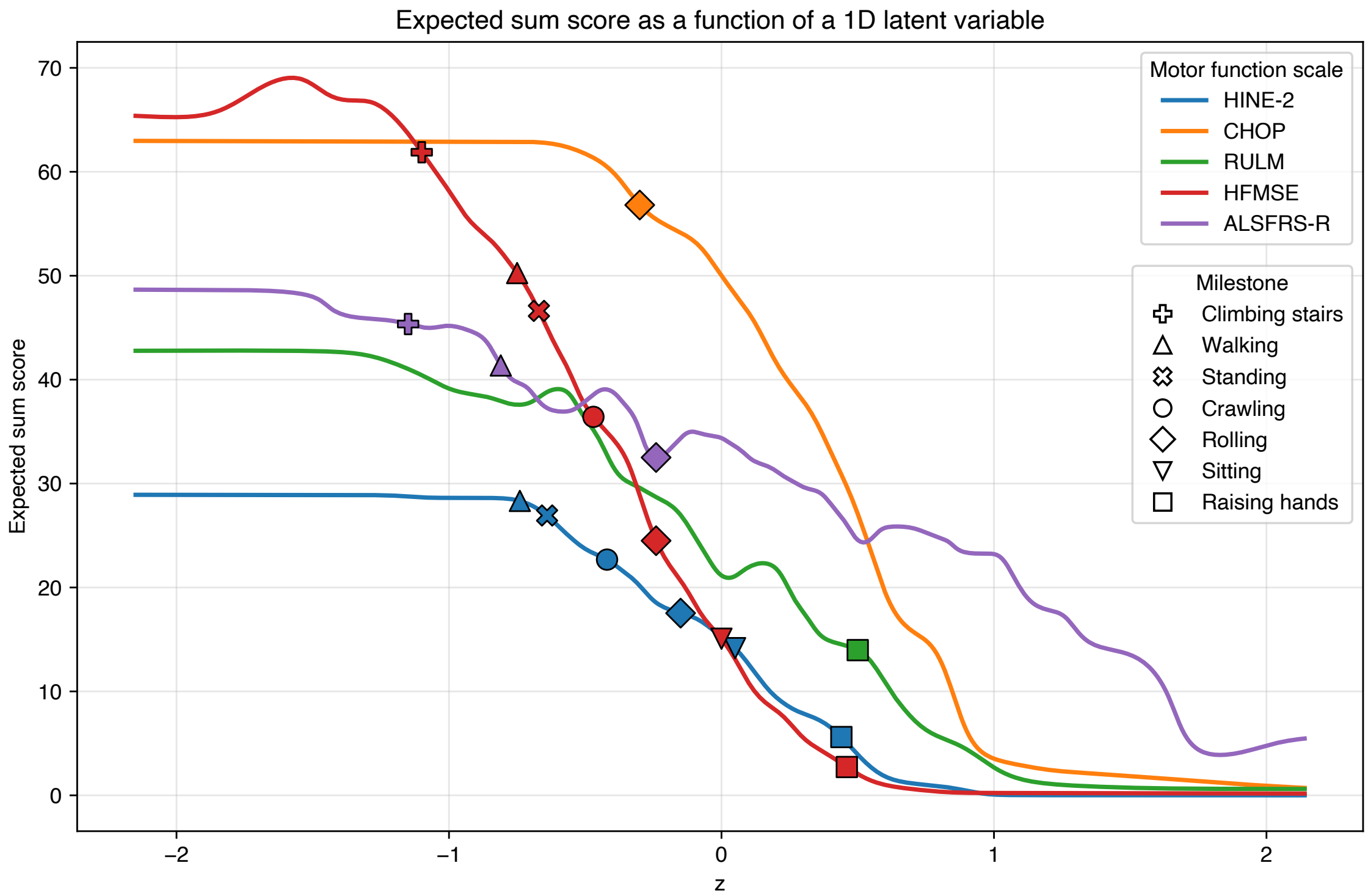

Figure S1: Latent alignment across five measurement instruments. In a one-dimensional latent space, we decoded z-values of a one-dimensional model using each of the five instruments and plotted the expected sum scores of the resulting decoder distributions (see Figure 1). For each instrument, we marked attainment of motor-function milestones when more than 50% of decoded samples achieved the respective milestone. Because items do not correspond one-to-one across instruments, we manually matched similar motor function items while disregarding slight deviations in their meaning. For instance, "rolling" in the ALSFRS-R test reflects the ability to turn and roll in bed, whereas in HINE-2 it encompasses rolling from prone to supine and vice versa over one side, and in the HFMSE test it encompasses being able to roll from supine to prone and vice versa over both sides. Overall, we observe a consistent alignment of motor-function milestones across the different measurement scales.

	Real			Reconstruction error		
Instrument	Mean	SD	Percentage	Mean	SD	Percentage
$d = 1$			1.84			8.02
HINE-2	0.59	0.22	2.03	2.79	0.43	9.62
RULM	1.14	0.22	2.65	3.99	0.49	9.27
CHOP	1.49	0.26	2.32	6.36	1.57	9.93
HFMSE	1.47	0.21	2.04	4.55	1.00	6.32
ALSFRS-R	0.41	0.17	0.79	2.59	0.46	4.98
$d = 2$			2.35			6.41
HINE-2	0.81	0.31	2.79	2.16	0.48	7.44
RULM	1.43	0.22	3.32	2.91	0.77	6.77
CHOP	1.15	0.38	1.80	5.51	1.28	8.60
HFMSE	1.68	0.38	2.33	3.92	1.02	5.44
ALSFRS-R	0.80	0.59	1.54	1.98	0.60	3.80
$d = 3$			2.20			5.36
HINE-2	0.87	0.20	3.00	1.86	0.37	6.41
RULM	1.47	0.12	3.42	2.36	0.64	5.48
CHOP	0.91	0.31	1.42	4.79	1.00	7.48
HFMSE	1.56	0.38	2.16	3.23	0.77	4.49
ALSFRS-R	0.52	0.30	1.00	1.52	0.58	2.92

Table S1: Reconstruction error statistics and estimated treatment switch effects for choices of the latent dimension $d \in \{1, 2, 3\}$. For smaller values of d, reconstruction quality, i. e. the difference in test sum scores, decreases noticeably, while the estimated treatment effect remains unchanged in direction but becomes slightly smaller in magnitude.

References

1. Nazábal A, Olmos PM, Ghahramani Z, and Valera I. Handling incomplete heterogeneous data using VAEs. Pattern Recogn 2020; 107:107501. DOI: 10.1016/j.patcog.2020.107501
2. Kingma DP and Ba J. Adam: a method for stochastic optimization. arXiv preprint 2014. DOI: 10.48550/arXiv.1412.6980
3. Liu DC and Nocedal J. On the limited memory method for large scale optimization. Math Program 1989; 45:503–28. DOI: 10.1007/BF01589116
4. Levina E and Bickel PJ. Maximum likelihood estimation of intrinsic dimension. *Advances in Neural Information Processing Systems*. 2005

5. Facco E, d'Errico M, Rodriguez A, and Laio A. Estimating the intrinsic dimension of datasets by a minimal neighborhood information. Scientific Reports 2017; 7:12140
